# Semi-quantitative, spatially resolved analysis of protein deposit layers on membrane surfaces

**DOI:** 10.1016/j.mex.2019.100780

**Published:** 2019-12-19

**Authors:** Martin Hartinger, Jonas Napiwotzki, Eva-Maria Schmid, Franziska Kurz, Ulrich Kulozik

**Affiliations:** Chair of Food and Bioprocess Engineering, Technical University of Munich, Germany

**Keywords:** Semi-quantitative, spatially resolved analysis of protein deposit layers on membrane surfaces, Deposit layer, Spatially resolved analysis, Visualization, Quantification, Protein, Skim milk, Staining, Coomassie Brilliant Blue, SWM, Polymeric membrane

## Abstract

Fouling distinctly reduces the filtration performance of membranes. A characterization of the fouling in membranes, however, is difficult due to its spatial distribution. Currently applied methods for deposit layer analysis are rather complex or do not offer a spatial resolution. Knowledge of the spatial distribution, however, could be used to improve the design of membranes, modules, and spacers. Staining with Coomassie Brilliant Blue, related to the staining of PAGE gels, is a simple method to visualize and analyze the deposited proteins semi-quantitatively. We improved an existing staining technique for protein deposits on membranes by adding a calibration for the semi-quantitative analysis and optimizing the sample handling. The method provides a spatially resolved analysis of deposited proteins up to a concentration of 10 g m^−2^. Apart from staining, data processing is described in order to generate false colors or topographic images of deposits. Thus, the paper describes a simple method to assess and visualize the influence of module characteristics such as spacer design on the spatially resolved protein fouling of polymeric and ceramic membranes. Therefore, the method can contribute to the improvement of the module design and processing conditions with regard to the filtration performance.

• Visualization of proteinaceous deposits on membranes
• Spatially resolved quantification of proteinaceous deposits

## Graphical abstract

**Figure:**
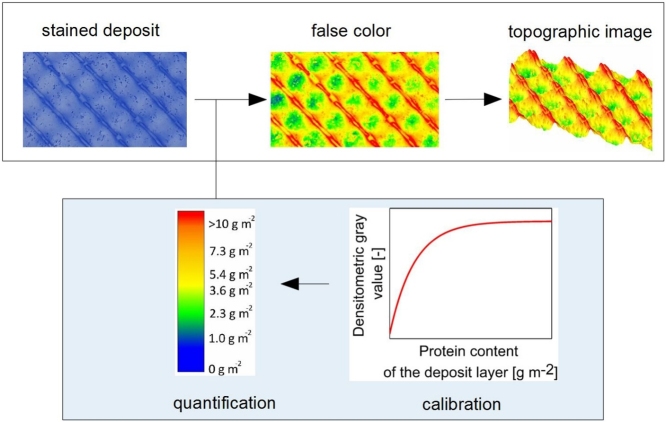

**Specification Table**

**Table:** 

Subject Area:	Chemistry
More specific subject area:	Membrane filtration
Method name:	Semi-quantitative, spatially resolved analysis of protein deposit layers on membrane surfaces
Name and reference of original method:	Reisterer, K. M.; Zottola, E. A.; Rulcher, R. G. [1]: Mapping protein foulants on polysulfone membranes using microspectrophotometry. In: Food technology 47, S. 104–108.
Resource availability:	• Coomassie Brilliant Blue R250 (PhastGel Blue, GE Healthcare Bio-Sciences, Chalfont St Giles, Great Britain) • ethanol 99.5 % • acetic acid • paper filter (Grade 595 ½, Whatman International Ltd, Maidstone, England) • gray reference plate (for example cut from a piece of plastic) • desiccator • Gel Doc XR+ (Bio-Rad Laboratories, Inc., Hercules, USA) for image generation • Dead end filtration test cell • Reversed-phase high performance liquid chromatography (RP-HPLC) • Guanidine buffer (guanidine concentration 6 M) (according to Dumpler et al. [2]) • Image Lab (Version 3.0.1 or later) • ImageJ (1.5.1f or later) for image analysis and false color generation

## Method details

### Background information and applicability of the method

The method is based on the staining of proteins by Coomassie Brilliant Blue (CBB) R250 (reddish). Apart from CBB R, a greenish CBB G exists, which contains two additional methyl groups [3]. CBB R is mainly used for the staining of proteins in gels such as used in polyacrylamide gel electrophoresis (PAGE), whereas CBB G is mainly applied for the quantification of proteins in solutions [4], e.g., in the Bradford assay [5]. According to Tal et al. [3], CBB R binds to basic amino acids via electrostatic interactions. Hydrophobic interactions with amino acids adjacent to the basic amino acids are likely to take place and enhance the amount of bond CBB. Other amino acids, especially acidic amino acids such as glutamic acid were found not to interact significantly with CBB. Apart from proteins, CBB does not show binding affinity to many other substances [6]. Therefore, the protein content of a sample can be correlated with the amount of CBB bond after staining.

Kain and Henry [7] described a method for the quantification of proteins by CBB via absorbance at 595 nm after blotting proteins from a SDS (sodium dodecyl sulfate)-PAGE gel on a polyvinylidene fluoride (PVDF) membrane and staining. They showed a linearity of the absorbance for protein bands from 0.5–10 μg. However, they did not quantify the proteins directly on the membrane but eluted the bond CBB from the membrane and measured the absorbance of the eluate. In contrast to that, Reisterer et al. [1] showed that CBB can be used to stain and analyze protein fouling directly on polysulphone (PSU) membranes. When virgin membranes were used, a low amount of CBB was present on the membrane after staining and destaining. This could be attributed to entrapment of CBB molecules in the membrane or a slight interaction of the membrane with CBB. Reisterer et al. [1] showed that the different amounts of protein on the membrane resulted in different extents of bond CBB. However, they did not correlate the absorbance with the amount of deposited protein, thus, no quantification of the deposited protein was carried out.

In this regard, we improved the method of protein staining for membrane foulants by correlating the CBB color intensity on the fouled membrane to the amount of area specific protein fouling on the membrane. The color intensity of the fouled membrane was converted into a black and white image. The gray value of the image was then correlated to the protein content of the membrane. Areas with more intense blue (darker areas in the black and white image) held higher contents of protein.

In contrast to Reisterer et al. [1] but in accordance to Kain and Henry [7], PVDF was used as membrane material in the method development. The method was calibrated and validated for skim milk derived deposits. Nevertheless, the method is likely to be also applicable to proteinaceous foulants other than skim milk proteins. However, the binding capacity of CBB differs among proteins [6,7]. Therefore, new calibration curves have to be generated. Since other components of the fouling layer except for proteins could bind CBB or proteins could be entrapped within other foulants and thus be no accessible to interactions with CBB, the method has to be validated for applications other deposits than skim milk proteins.

Regarding the membrane material, polyethersulphone (PES), polyvinylpyrrolidone (PVP)/PSU blend, polyamide (PA), and titanium oxide did not show any interaction with CBB. PVDF, PSU, aluminum oxide, and zirconium oxide showed a slight interaction with CBB, which resulted in a slight bluish color of the virgin membranes (no protein adherent to the membrane) after staining. Since the blank value of the membrane, generated from staining a virgin membrane, is subtracted from the measured value, this does not affect the accuracy of the results. Thus, other membrane materials than PVDF and PSU can possibly be used with the staining method, which, however, has to be tested in detail. It has to be noted that the membrane materials have to be resistant to the solutions used in this method.

With this method, deposits can be quantified directly on the membrane after filtration without any blurring of the deposit pattern. This means, the deposit can be assessed spatially resolved along an individual pattern such as a grid or a line pattern. This is an advantage to quantification methods of protein fouling, which need to dissolve proteins from the membrane [[7], [8], [9]]. Confocal laser scanning microscopy (CLSM) can also be applied for the quantification of proteins in deposits on the membrane. To do so, a labeling of proteins has to be carried out before the filtration [10,11]. However, protein fouling properties can change upon labelling [12]. Although, the protein content in the deposit can be estimated by CLSM, no exact quantification is possible, which is also a limitation of the ultrasonic deposit layer analysis [13]. Since the staining method is capable of quantifying deposited proteins directly on the membrane, it is a cost-efficient and easy analysis for the spatially resolved deposition of proteins on membranes.

### Preparation of the staining solution and deposit layer staining

1 Mix 250 ml ethanol and 80 ml acetic acid and fill up to 400 ml with deionized water
2 Dissolve a pellet of Coomassie Brilliant Blue R250 in the solution
3 Heat the solution to 60 °C under continuous stirring
4 Cool the solution to 20 °C and store it for 12 h
5 Filter the solution with a paper filter Grade 595 ½ (Whatman International Ltd)
6 Dilute the solution: add 3 parts of ethanol to 2 parts of the solution. The diluted solution was found to result in the best resolution of the staining technique.

### Membrane preparation

1 Extract membrane directly after filtration
2 Immerse the membrane in deionized water (20 °C) for 30 s to remove residual feed
3 Dry the membrane in a desiccator (at least 30 min)

*Note:* Freely attached water must not be present anymore. The deposit is just loosely bond to the membrane surface. When the membrane is immersed in the staining solution without drying, the deposit layer likely detaches from the membrane and cannot be analysed anymore. The membrane must not dry out completely either, since the deposit layer could crack and results would be negatively affected.

### Staining

1 Cover the membrane sample with the diluted staining solution
2 Stain for 10 min at room temperature under gentle shaking (8 rpm, angle of 10°)
3 Destain the membrane in sufficient amounts of ethanol (ethanol level at least 2 cm) for 3 min

*Note*: Ethanol hinders the dissolution of proteins from the deposit into the staining and destaining solution in contrast to water. Thus, the membrane must not be washed with water before the destaining step. This would cause the deposits fixed to the membrane by the ethanol of the staining solution to dissolve in the washing water.

4 Air-dry the membrane for at least 30 min at room temperature

*Note:* The color of the stained membrane will brighten up with drying and reach a steady state color (compare Fig. 4). Therefore, a sufficient drying of the sample before analyzing is mandatory. During drying, the membrane might coil. Thus, weighting of the membrane during drying is required.

### Sample imaging

1 Pre-warm the light source of the gel scanner for at least 30 min to obtain a constant luminance

*Note*: A lower light intensity (e.g., right after starting up the light source) would cause higher gray values and shift the results toward higher deposit concentrations.

2 Ensure that the dried membrane samples are lying in the Gel Scanner without coiling to avoid shadows
3 Insert a reference plate to verify correct luminance of the light source; the reference plate can be cut from a piece of plastic and has to be analysed with every membrane sample. The gray value of the reference should be in the calibration range of the method.
4 Take a black and white image of the membrane sample and the reference plate (exposure time 0.6 s)
5 Check, if the reference plate has the same gray value in each image to ensure the same luminance was reached.

*Note*: The luminance affects the gray value and therefore the detected amount of deposited protein. A variation in the gray value of the reference plate indicates measurement errors due to altered illumination.

### Image analysis (screen shots showing the prompts are provided in the supplementary)

1 Export the received image for analysis in .tiff format with Image Lab to ensure a high resolution
2 Import the image into ImageJ (Version 1.51f or later) via drag and drop with a 16-bit gray scale.
3 Proceed with **A**, **B**, or **C** in order to obtain the local content or spatial distribution (**A**), a false color (**B**), or a topographic image (**C**) of the deposit

**A:** Local or spatial resolved deposit layer content

*Note*: With A, the local gray values of the membrane can be evaluated with an arbitrary pattern such as a grid pattern and be correlated to the amount of deposited protein

1 Select the parts of the image to be analyzed, e.g., for the deposit layer distribution along a line parallel or orthogonal to a spacer strand use the line tool to draw a line on the specific part of the image or combine several lines to obtain a grid or line pattern for spatially resolved information on the deposit layer. Alternatively, single spots can be chosen (Fig. 8 in Supplementary data)
2 Open the region of interest (ROI) manager (analyze – tools – ROI-manager) (Fig. 9 in Supplementary data)
3 Add the selection by the button Add (Fig. 10 in Supplementary data)
4 Plot the Multi Plot (in the ROI-manager: More – Multi Plot) (Fig. 11 in Supplementary data)
5 Copy data set (in the Multi Plot: More – Copy 1^st^ data set) to a calculation program (Fig. 12 in Supplementary data)
6 Convert the gray values to the deposit layer concentration by the calibration curve

**B:** False color

*Note*: With B, the black and white image can be converted into a false color displaying the different protein concentrations on the membrane at different colors.

1 Open the Lookup table (LUT) tool (Image – Color – Edit LUT) (Fig. 13 in Supplementary data)
2 Load a Lookup table (Open) (Fig. 14 in Supplementary data)
  *Note*: A LUT displaying the range between 0 and 10 g m^−2^ in a color sequence from blue to red can be found in the supplementary.
3 Adjust minimum and maximum brightness value to 0 and 4095, respectively (Fig. 15 in Supplementary data)
  *Note*: Use a plugin like “Set min and max values for LUT (v1.00)” [14]. Since some images do not have a minimum and maximum brightness of 0 and 4095, respectively, equal amounts of the deposited protein would otherwise result in different colors in the different pictures.
4 Add calibration bar (Analyze – Tools – Calibration Bar) (Fig. 16 in Supplementary data)

**C:** Topographic image

*Note*: With C, topographic images of the deposit layer distribution on the membrane can be produced. The black and white image is converted to a false color like in B.

1 Start as described in **B:** False color (up to **3**)
2 Open the Interactive 3D surface plot (Plugins – 3D – Interactive 3D surface plot) (Fig. 17 in Supplementary data)
3 Adjust the properties of the plot

### Calibration of the staining method

1 Produce homogeneous deposit layers with varying protein concentrations in the dead-end test cell
2 Cut the membrane in halves of the same size
3 Stain half 1 and process according to Image analysis in order to receive the gray value of the stained membrane
4 Immerse half 2 in guanidine buffer (guanidine concentration 6 M) (according to Dumpler et al. [2]) for 60 min at room temperature to dissolve the deposit layer completely
5 Analyze the quantity of deposited proteins by RP-HPLC according to Dumpler et al. [2]. The operating conditions of the RP-HPLC are described in detail by Dumpler et al. [2].
6 Correlate the gray value with the deposit layer quantity

## Method validation

### Production of homogeneous deposits

**Fig. 1:**
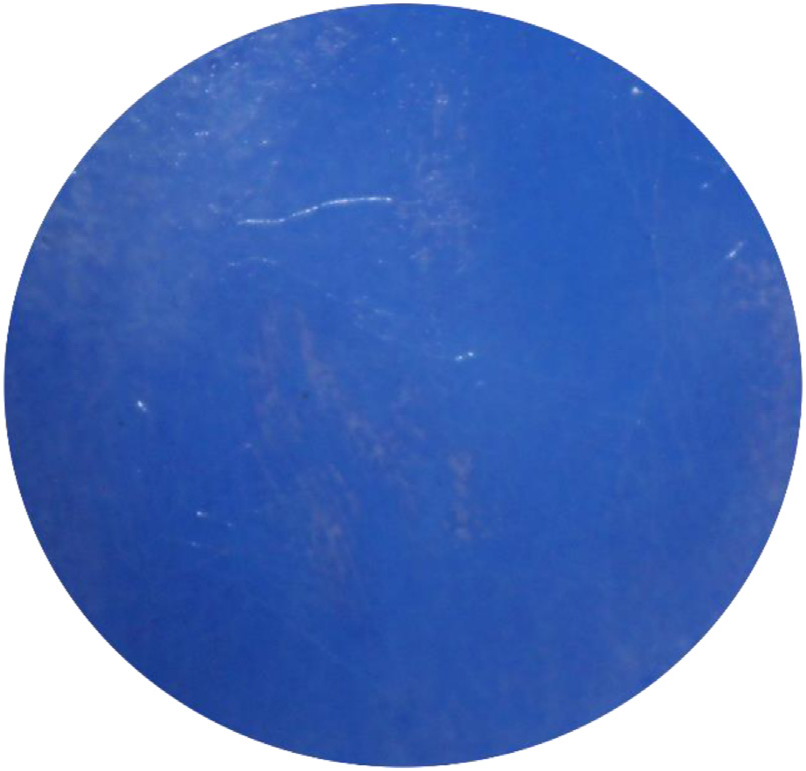
Stained deposit layer after a filtration with 0.5 bar transmembrane pressure for 2 min.

Staining by Coomassie Brilliant Blue (CBB) is initially time dependent. Therefore, its staining kinetics for deposited proteins had to be investigated. For this analysis, a homogeneously distributed deposit layer was mandatory. The deposit was produced by dead-end filtration of skim milk at 20 °C at a transmembrane pressure of 0.5 bar and 2 min filtration time using a dead-end test cell (AMICON 8050, Merck-Millipore, Billerica, MA, USA). Fig. 1 shows the deposit layer distribution after dead-end filtration and staining. Thereby, the color intensity of the membranes is related to the deposition. A more intense blueish color indicates more intense protein fouling. The homogeneous distribution of the blue color on the membrane surface confirms the uniform distribution of the produced deposit layer. It has to be noted that the color intensity did not exceed the saturation limit of the method (compare Fig. 5)

### Deposit layer staining, destaining, and drying

**Fig. 2:**
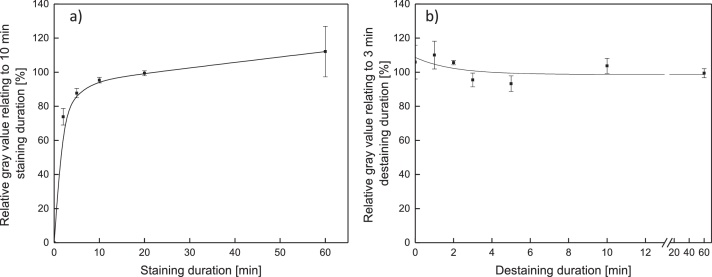
Relative gray value (as a ratio to a sample stained for 10 min (a) and destained for 3 min (b)) as a function of the treatment duration. The deposition of protein for the staining and destaining time tests was between 2 and 6 g m^−2^.

For the investigation on the deposit layer staining time, a membrane with a homogeneous deposit layer was divided in two parts after filtration of skim milk and then dried in a desiccator to remove adherent water. Afterwards, one half was stained for 10 min (as reference), whereas the staining time of the other half was varied (Fig. 2a). For the same staining time (both halves stained for 10 min), the relative color intensity is close to 100 %. This proves that the deposit layer was homogenously distributed on the membrane sample and both halves contain the same amount of deposited protein. A difference in the gray value of both halves is therefore caused by the influence of the exposure time of the deposit to the staining solution. It can be seen that the color intensity increases strongly within the first 5 min. After this initial phase, the increase flattens but no asymptotic behavior could be observed within 60 min. Besides the staining time, the destaining time was investigated the same way with a destaining time of the reference of 3 min. The gray value plateaued almost instantly and was constant for at least 60 min (Fig. 2b). To keep the method as short as possible, destaining time was set to 3 min.

**Fig. 3:**
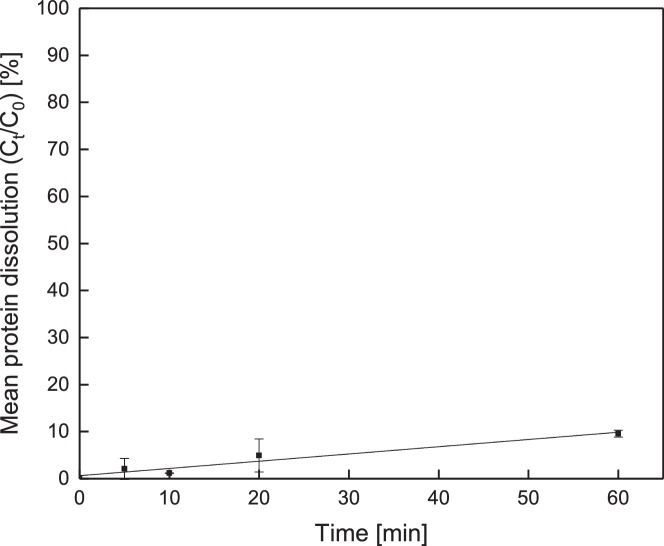
Proportion of deposit layer dissolved in the staining solution as a function of the staining time (concentration of deposited protein: 36.67 g m^−2^ ± 2.37 g m^−2^, staining solution 5 mL).

It seems reasonable to stain membranes for at least 10 min. With a lower staining duration, time dependency of staining is more distinct and reproducibility decreases. On the other hand, a longer staining time causes dissolution of protein into the dye solution (Fig. 3). The desorption of the deposit layer into the staining solution was investigated at a deposition of about 36.7 g m^−2^ (beyond the saturation limit of the staining method) to ensure a concentration of dissolved protein in the supernatant above the detection limit of the RP-HPLC. The proportion of protein dissolved in the dye solution increases linearly with time. At 60 min, 10 % of the deposited protein were dissolved. In this regard, staining time has to be as low as possible. Referring Fig. 2, 10 min is sufficient to ensure good reproducibility of staining and keeping dissolution at a minimum value.

**Fig. 4:**
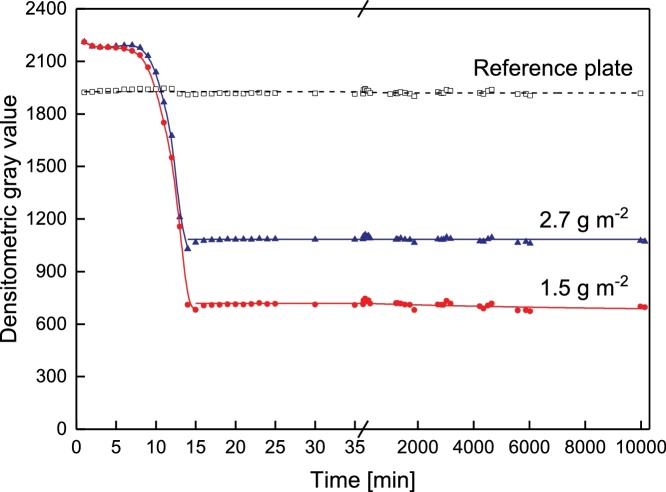
Color changes upon drying and aging. Deposit layers had a mean protein content of 2.7 g m^−2^ and 1.5 g m^−2^, respectively.

**Fig. 5:**
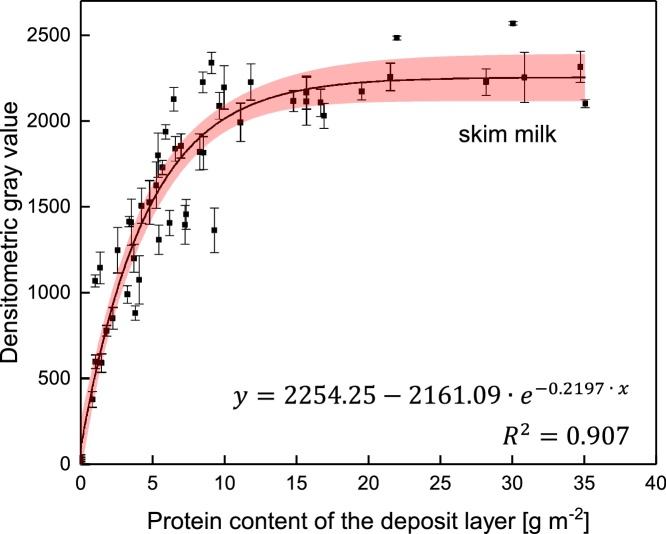
Color intensity of CBB stained deposits as a function the protein quantity. Standard deviation shows the deviation in the densitometric gray value of three measurement points on the membrane with a size of 76 mm^2^ each. The red band shows the 95 % confidence interval.

After destaining, the membrane has to dry, as otherwise color changes affect the quantification. Independent of the amount of protein on the membrane, color intensity decreased within the first 15 min (Fig. 4). Thereafter, no change in intensity could be found for a period of 7 days as long as the membrane was stored in the dark.

Staining and gray value analysis can therefore be performed at different days. To sum up, a staining time of 10 min and a destaining time of 3 min showed good reproducibility and accuracy. After destaining, the membrane has to be dried for at least 15 min in order to receive constant color intensity values. However, we propose a drying time of 30 min.

### Calibration of the staining method

As it is known for SDS-PAGE gels, protein contents can be determined semi-quantitatively via photometric absorbance. In this method, the amount of deposited proteins on the membrane surface should be determined after staining. Since Reisterer et al. [1] reported a slight color response of virgin PSU membranes without proteins, we stained virgin PVDF membranes without protein in order to receive a blank value. The blank gray value of stained virgin PVDF membranes was found to be 1251.5. This blank value was subtracted from all measured values to receive the color intensity of CBB related to protein fouling.

For the calibration of the staining method, a dead-end test cell produced deposit layers with different protein contents by filtering skim milk. Since the depositions were homogeneous (compare Fig. 1), one part could be used for the quantification via RP-HPLC, whereas the other part could be stained.

The correlation between color intensity of the CCB stained deposits (blank value of 1251.5 subtracted from all measured values) and the quantity of protein (via RP-HPLC) is shown in Fig. 5 for deposits on a 0.1 μm PVDF membrane (Synder Filtration, Vacaville, CA, USA). The color intensity increases with the protein content on the membrane until a maximum value of about 2270 is reached. The independence of the color intensity from the protein content at values higher than 15 g m^−2^ is due to saturation effects. With a protein content of <10 g m^−2^ a distinct differentiation in color intensity as function of deposit layer content can be observed as the correlation function is distinctly rising. Therefore, 10 g m^−2^ is the maximum detectable value of the method. According to the 95 % confidence interval, the minimum distinguishable value of deposited protein is 0.4 g m^−2^.

Depending on the filtration conditions, the protein composition of the deposit layer varies [15]. For protein solutions, Bradford [5] reported a scattering in the absorbance at 595 nm for different protein species. Compton and Jones [6] showed that CBB G binding properties are dependent on the amino acid profile of the protein. It was found that the response of CBB G binding is thus dependent on the specific protein [16]. Tal et al. [3] found differences in binding properties of CBB R (reddish) to proteins in SDS-PAGE gels as well.

Thus, an investigation on the color responses of CBB to the different proteins present in skim milk deposited on a membrane surface is of particular interest. In order to assess the CBB binding capacity of different protein species in the deposit layer, the casein fraction, the major whey protein β-lactoglobulin (β-lg), and the total whey protein fraction were investigated.

Relating to the mass, the casein fraction contains more arginine (25 %) and histidine (15 %) but less lysine (25 %) compared to the whey protein fraction (in this case whey protein isolate (WPI)) [17]. The major whey protein β-lg possesses slightly less arginine (8 %), about half the amount of histidine, but more lysine (14 %) compared to the casein fraction [18]. Since basic amino acids were found to be the main binding sights of CBB [3], a variation in the amino acid profile might influence the color response of the deposited proteins after staining. Therefore, the color response of deposited micellar casein, WPI, and β-lg was investigated. Micellar casein was derived from microfiltration of skim milk. Milei (Leutkirch, Germany) manufactured the WPI. The β-lg was produced by a method described by Toro-Sierra et al. [19] from whey protein isolate. In order to deposit higher amounts of β-lg on the membrane surface, β-lg particles with a bigger size than the native β-lg were formed. Therefore, a 1.0 % β-lg solution in deionized water was treated at 80 °C for 90 min at a pH of 5.8. This resulted in particles with a size of about 0.1 μm. The isolated protein fractions were used to create solutions with concentrations ranging from 1 to 10 % (dilution in milk serum), which were then filtrated to generated deposits of varying protein contents.

**Fig. 6:**
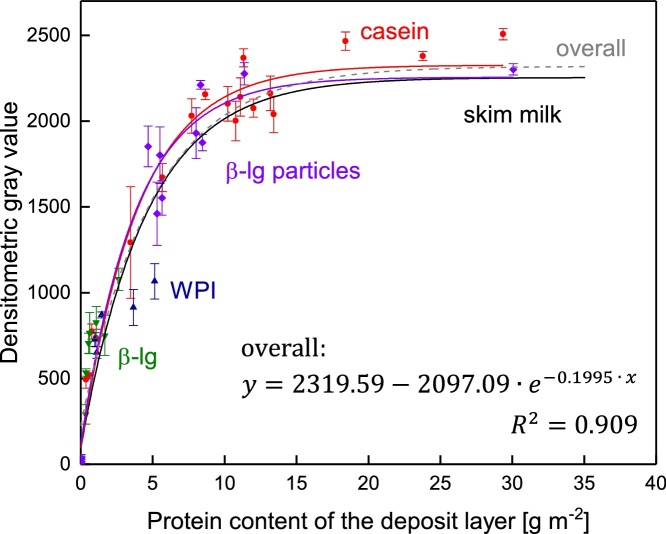
Color intensity of CBB stained deposits of varying protein sources as a function the protein quantity (skim milk data points were removed for better clarity). Standard deviation shows the deviation in the densitometric gray value of three measurement points on the membrane with a size of 76 mm^2^ each.

Fig. 6 shows the color responses of the main proteins and fractions of skim milk (casein, β-lg, and WPI) as a function of their concentration on the membrane. It is observable that no relevant differences in the color response occur. For caseins, WPI, and β-lg deposited on the membrane surface, the color intensity is similar to that of skim milk. Hence, variances in the deposit layer´s protein composition with varying filtration conditions can be neglected in terms of determining the spatially resolved total protein content.

### Spatial resolution of the staining method

**Fig. 7:**
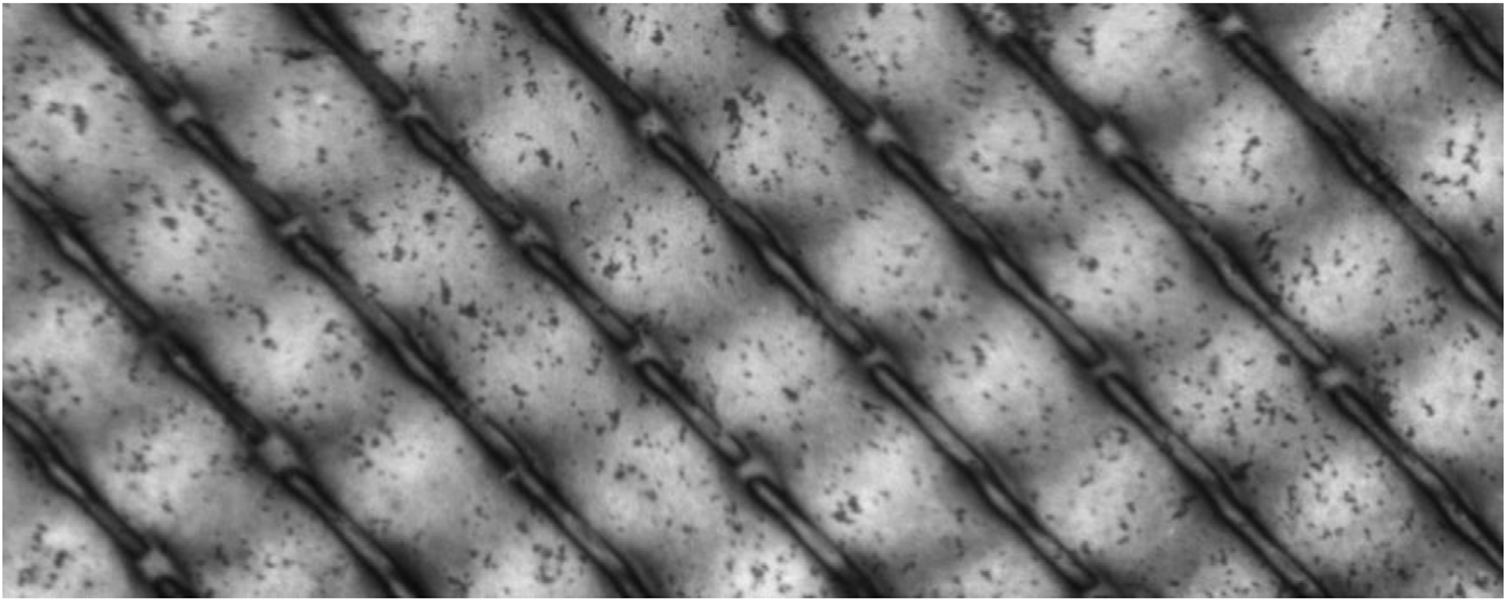
Membrane with stained deposit layer. The deposit layer pattern was generated by a 44 mil diamond spacer. Filtration conditions: 0.5 bar transmembrane pressure; 1.0 bar m^−1^ axial pressure drop; skim milk CF 3; 10 °C. The mean deposit layer content was 5.3 g m^−2^ (by RP-HPCL) with a maximum local concentration >10 g m^−2^.

The main purpose of the staining method is to obtain amounts of deposit layer spatially resolved. This means, the deposit layer pattern should be assessed along the longitudinal and transverse axis or any other axis system. To do so, it was to investigate, if the deposit layer patter was blurred by the staining technique. Therefore, we stained a membrane after filtration in a test cell with a spacer filled channel [20] to obtain a pattern with sharp edges in protein concentration on the membrane. The filtration was carried out with ultrafiltrated skim milk (concentration factor (CF) 3, i.e., the same serum composition as skim milk but a 3-fold concentrated protein phase). The filtration was carried out for 60 min with the 0.1 μm PVDF membrane used to generate the calibration with a 44 mil diamond spacer at 0.5 bar transmembrane pressure and 1.0 bar m^−1^ axial pressure drop at a temperature of 10 °C. The membrane was extracted from the test cell immediately after filtration and afterwards treated according to this method´s protocol. The membrane after the staining procedure is depicted in Fig. 7 in a black and white image taken with the gel scanner. It can be seen that the spacer induces areas of high deposition in close vicinity to areas of almost no deposition. Furthermore, it can be seen that the edges are not blurred. Lines of a width of 100 μm could be resolved with the staining method (assessed by a reflecting microscope image of the stained membrane). Thus, the staining method can be used to obtain data to determine the spatial distribution of proteins on the membrane. For obtaining spatial resolved data from the stained membranes after generating black and white images, image evaluation according to A (“local or spatial resolved deposit layer content”) can be used.
